# Supplementary material for: Thriving in a pandemic: Determinants of excellent wellbeing among New Zealanders during the 2020 COVID-19 lockdown; a cross-sectional survey
Source: PLoS One. 2022 Mar 3;17(3):e0262745. doi: 10.1371/journal.pone.0262745 (PMC8893611; doi:10.1371/journal.pone.0262745)
Supplement: S1 File — (PDF) [file pone.0262745.s001.pdf]

Lockdown Wellbeing Survey

Start of Block: 1. Introduction

Q1.1 **Kia Ora and Welcome**

Thank you for clicking through to our survey; it should take you around 10 to 15 minutes to complete. The survey is being conducted by researchers from the University of Otago.

To go directly to the survey please click on the 'Next' button at the bottom of the page

If you lose your connection to the Internet or this survey at any point, please click the link provided in the email you received and it will take you back to the point where you left off.

Q115 **To complete this survey, you must be 16 years old or older.**

Are you 16 years old or older?

- ○ Yes (1)
- ○ No (2)

Skip To: End of Survey If To complete this survey, you must be 16 years old or older. Are you 16 years old or older? = No

Page Break

Q1.2

THE EFFECTS OF COVID-19 AND THE LOCKDOWN ON WELLBEING IN NEW ZEALAND

Information for participants

What will participants be asked to do? The COVID-19 pandemic has resulted in major disruptions to our lives. Research from overseas has shown that pandemics and lockdowns have significant effects on people's well-being and mental health, but we do not yet have similar research from Aotearoa New Zealand. We are interested in the experience of New Zealanders and this is what we would like to ask you about. Should you agree to take part in this survey, you will be asked about your experiences over the course of the COVID-19 lockdown, including how it has affected your employment, your mental health and well-being, your behaviours, and any 'silver linings' or positive experiences. The survey should take about 10 to 15 minutes to complete.

Thank you for showing an interest in this study. Please read this information sheet carefully before deciding whether or not to participate. If you decide to participate, we thank you. If you decide not to take part there will be no disadvantage to you and we thank you for considering our request. **What is the aim of the project?** This study explores the experiences of the New Zealand population during the current COVID-19 event, including the subsequent imposed social isolation measures (the Level 4 'lockdown'). **What types of participants are being sought?** We are seeking adult participants from the general population aged 16 years and older. We are applying quotas so we ensure our sample has reasonable numbers of people from different ethnicities, and roughly similar numbers across genders. **What data or information will be collected and what use will be made of it?** All your answers will be completely anonymous to the research team. Results will be shared with The Ministry of Health and Ministry of Justice. The results of the project may also be published and will be available in the University of Otago (New Zealand) library; however there will be no way to trace responses back to individuals therefore the anonymity of participants will be preserved. The results will also be shared with the Ministry of Health to inform the support packages they offer in response to the COVID-19 crisis. The data collected will be securely stored in such a way that only members of the research team will be able to gain access to it. Data obtained as a result of the research will be retained for at least five years in secure storage. Any information held may be destroyed after five years even though the data derived from the research will, in most cases, be kept for much longer or possibly indefinitely. **Can participants change their mind and withdraw from the project?** You may withdraw from participation in the survey at any time and without any disadvantage to yourself. **What supports are available?** Some of the questions are about potentially sensitive topics like suicidal thoughts or family violence. We are asking about these topics because overseas evidence has suggested changes in their frequency related to the COVID-19 crisis. Like all other questions, your response to these questions is anonymous. You do not have to answer any question you do not wish to. If you feel negatively affected thinking about any of these topics, please use the contact details for the support services

provided or free call or text 1737 any time, 24 hours a day to talk to a trained counsellor. This service is completely free. **What if participants have any questions?** If you have any questions about our project, either now or in the future, please feel free to contact: Dr Matthew Jenkins
Department of Psychological Medicine, University of Otago, Wellington Email Address: matthew.jenkins@otago.ac.nz This study has been approved by the University of Otago Human Ethics Committee (reference F20/003). However, if you have any concerns about the ethical conduct of the research you may contact the University of Otago Human Ethics Committee (Gary Witte: gary.witte@otago.ac.nz or Jo Farron de Diaz: jo.farrondediaz@otago.ac.nz). Any issues you raise will be treated in confidence and investigated and you will be informed of the outcome.

Page Break

Q1.3 As part of the University of Otago's ethics approval process we need to ask you to answer the following question before starting our survey.

I have read the Information Sheet for this project and agree to take part in the study.

○ Yes (1)

○ No (2)

Skip To: End of Survey If As part of the University of Otago's ethics approval process we need to ask you to answer the fol... = No

End of Block: 1. Introduction

Start of Block: 2. Ethnicity

Q2.3 **Which of these ethnic groups do you identify with?**

Please select ALL the ethnic groups that you identify with.

- ☐ New Zealand European (1)
- ☐ Māori (2)
- ☐ Samoan (8)
- ☐ Cook Island Māori (9)
- ☐ Tongan (4)
- ☐ Niuean (10)
- ☐ Chinese (6)
- ☐ Indian (5)
- ☐ Other (e.g. Dutch, Japanese, Tokelauan). Please state: (7) ____

Page Break

Display This Question:

If Which of these ethnic groups do you identify with? Please select ALL the ethnic groups that you... = Māori

Q2.4 **Do you belong to any local iwi close to where you live?**

- ◯ Yes (1)
- ◯ No (2)
- ◯ Don't know (3)

Display This Question:

If Which of these ethnic groups do you identify with? Please select ALL the ethnic groups that you... = Māori

Q2.5 **Thinking about your connection to Māori culture, in the last 12 months, but prior to lockdown, did you do any of these things?**

Please tick all that apply

- ▢ Went to a marae (3)
- ▢ Went to a Māori festival (e.g., Pā Wars. Matariki, or Waitangi Day) (2)
- ▢ Sang a Māori song, performed a haka, given a mihi or speech, or taken part in Māori performing arts or crafts (4)
- ▢ Took part in traditional Māori healing or massage. (5)
- ▢ ⊗None of these (6)

Display This Question:

If Which of these ethnic groups do you identify with? Please select ALL the ethnic groups that you... = Māori

Q2.6 **<u>In the last 12 months</u>, other than those listed in the previous question, are there any other important ways that you have connected to or expressed your Māori identity or heritage.**

- ○ Yes (please specify) (1) ____
- ○ No (2)
- ○ Don't know (3)

End of Block: 2. Ethnicity

Start of Block: Ethnicity

Q228 **Which of these ethnic groups do you identify with?**

Please select <u>ALL</u> the ethnic groups that you identify with.

- ▢ New Zealand European (1)
- ▢ Māori (2)
- ▢ Samoan (8)
- ▢ Cook Island Māori (9)
- ▢ Tongan (4)
- ▢ Niuean (10)
- ▢ Chinese (6)
- ▢ Indian (5)
- ▢ Other (e.g. Dutch, Japanese, Tokelauan). Please state: (7)

____

Page Break

Display This Question:

If Which of these ethnic groups do you identify with? Please select ALL the ethnic groups that you... = Māori

Q229 **Do you belong to any local iwi close to where you live?**

- ◯ Yes (1)
- ◯ No (2)
- ◯ Don't know (3)

Display This Question:

If Which of these ethnic groups do you identify with? Please select ALL the ethnic groups that you... = Māori

Q230 **Thinking about your connection to Māori culture, in the last 12 months, but prior to lockdown, did you do any of these things?**

Please tick all that apply

- ▢ Went to a marae (3)
- ▢ Went to a Māori festival (e.g., Pā Wars. Matariki, or Waitangi Day) (2)
- ▢ Sang a Māori song, performed a haka, given a mihi or speech, or taken part in Māori performing arts or crafts (4)
- ▢ Took part in traditional Māori healing or massage. (5)
- ▢ ⊗None of these (6)

Display This Question:

If Which of these ethnic groups do you identify with? Please select ALL the ethnic groups that you... = Māori

Q231 **<u>In the last 12 months</u>, other than those listed in the previous question, are there any other important ways that you have connected to or expressed your Māori identity or heritage.**

- ○ Yes (please specify) (1) ____
- ○ No (2)
- ○ Don't know (3)

End of Block: Ethnicity

Start of Block: 3. Living circumstances

Q3.1 **Your living circumstances**

These questions are about your living circumstances during the COVID-19 lockdown, which started on Thursday 26 March.

We define your 'bubble' as the household that you are in during the lockdown period, including anybody you are living with. Please note - this does not include people in other households, if you are living alone during lockdown.

Page Break

Q3.2 **<u>During the lockdown</u>, who has been living with you in your 'bubble'?**

- ◯ No one, I live by myself and have no pets (1)
- ◯ No one, I live by myself but I have one or more pets (2)
- ◯ Flatmates or tenants (3)
- ◯ Adult family/whānau member(s) only (4)
- ◯ Family/whānau members, including a child/children (8)
- ◯ Friends (5)
- ◯ A mixture of flatmates, family/whānau members or friends (6)

Display This Question:

If During the lockdown, who has been living with you in your 'bubble'? != No one, I live by myself and have no pets

And During the lockdown, who has been living with you in your 'bubble'? != No one, I live by myself but I have one or more pets

Q3.3 **Including yourself, how many people live with you in your bubble?**

____

Q3.4
**Not everybody has ended up in their bubble of choice.
How satisfied are you with the bubble you are in?**

- Extremely dissatisfied (27)
- Dissatisfied (28)
- Neither satisfied nor dissatisfied (29)
- Satisfied (30)
- Extremely satisfied (31)

Q3.5 **In your lockdown residence, which of the following are available to you?**

Please tick all that apply.

- Personal or quiet space (1)
- Internet (2)
- Computer (3)
- Easy access to a garden/green space (4)
- ⊗None of these (5)

Q3.6 **During the lockdown, how** easy has it been to stay connected with your family/whānau and friends outside your 'bubble'?

- ◯ Extremely hard (1)
- ◯ Somewhat hard (2)
- ◯ Neither easy nor hard (3)
- ◯ Somewhat easy (4)
- ◯ Extremely easy (5)
- ◯ I haven't tried to stay connected (6)

Display This Question:

If During the lockdown, how easy has it been to stay connected with your family/whānau and friends o... != I haven't tried to stay connected

Q3.7 During the lockdown, **how often have you connected each week with your family/whānau and friends outside your bubble?**

	**Not at all** (1)	**Once a week** (2)	**2-3 times a week** (3)	**4-6 times a week** (4)	**Every day** (5)
Talked in person (1)	◯	◯	◯	◯	◯
Talked by video (eg, Skype, WhatsApp) (2)	◯	◯	◯	◯	◯
Talked over the telephone (3)	◯	◯	◯	◯	◯
Connected by writing (text, email, snail mail) (4)	◯	◯	◯	◯	◯

Q3.8 **<u>Since the lockdown began,</u> how has your level of contact changed with family/whānau and friends outside your bubble?**

- ◯ It has increased (1)
- ◯ It has decreased (2)
- ◯ It has stayed the same (3)

Q3.9 **Overall, thinking about how well your family/whānau has been doing <u>since the lockdown</u>, would you say that things are currently getting better, getting worse, or staying about the same?**

- ◯ Getting better (1)
- ◯ Getting worse (2)
- ◯ Staying the same (3)

Display This Question:

If During the lockdown, who has been living with you in your 'bubble'? != No one, I live by myself and have no pets

And During the lockdown, who has been living with you in your 'bubble'? != No one, I live by myself but I have one or more pets

Q3.10 <u>During the lockdown</u>, **how are you and the people you are living with getting along with each other?**

- ◯ Very badly (1)
- ◯ Badly (2)
- ◯ Neither well nor badly (3)
- ◯ Well (4)
- ◯ Very well (5)

Q3.11 **<u>During the lockdown</u>, how often have you felt lonely or isolated?**

○ All of the time (1)

○ Most of the time (2)

○ Some of the time (3)

○ A little of the time (4)

○ None of the time (5)

Q3.12 **<u>During the lockdown</u> how much time have you been spending looking at information related to COVID-19?**

○ Less than an hour a day (1)

○ 1-2 hours per day (2)

○ 2-4 hours per day (3)

○ 4-8 hours per day (4)

○ More than 8 hours a day (5)

Page Break

End of Block: 3. Living circumstances

Start of Block: 5. New Employment

Q4.1

The following questions are about jobs and businesses during the COVID-19 lockdown.

Do you have a job now?

- ◯ Yes, I have a job (1)
- ◯ No, I don't have a job (2)
- ◯ I am self-employed (7)
- ◯ I am a business owner (9)
- ◯ I am retired (3)
- ◯ I have never had a job (10)

Skip To: End of Block If The following questions are about jobs and businesses during the COVID-19 lockdown. Do you ha... = I am retired

Skip To: End of Block If The following questions are about jobs and businesses during the COVID-19 lockdown. Do you ha... = I have never had a job

Page Break

Display This Question:

If The following questions are about jobs and businesses during the COVID-19 lockdown. Do you ha... = Yes, I have a job

Or The following questions are about jobs and businesses during the COVID-19 lockdown. Do you ha... = I am self-employed

Or The following questions are about jobs and businesses during the COVID-19 lockdown. Do you ha... = I am a business owner

Q4.2 **Have your hours of paid work been drastically reduced as a result of the lockdown?**

○ Yes (3)

○ No (5)

○ Doesn't apply to me (4)

Display This Question:

If The following questions are about jobs and businesses during the COVID-19 lockdown. Do you ha... = Yes, I have a job

Or The following questions are about jobs and businesses during the COVID-19 lockdown. Do you ha... = I am self-employed

Or The following questions are about jobs and businesses during the COVID-19 lockdown. Do you ha... = I am a business owner

Q4.3 Has your paid workload drastically increased as a result of the lockdown?

○ Yes (1)

○ No (2)

○ Doesn't apply to me (3)

Display This Question:

If The following questions are about jobs and businesses during the COVID-19 lockdown. Do you ha... = No, I don't have a job

Q4.4 **Have you lost your job (or jobs) as a result of the lockdown?**

- Yes (1)
- No (2)
- I didn't have a job before the lockdown (3)

Display This Question:

If The following questions are about jobs and businesses during the COVID-19 lockdown. Do you ha... = Yes, I have a job

Or The following questions are about jobs and businesses during the COVID-19 lockdown. Do you ha... = I am self-employed

Or The following questions are about jobs and businesses during the COVID-19 lockdown. Do you ha... = I am a business owner

Q4.5 **Are you an 'essential worker' (e.g., healthcare, law enforcement, emergency services, provider of essential goods)?**

- Yes (1)
- No (2)

Q4.6 **Is someone in your bubble an essential worker (e.g., healthcare, law enforcement, emergency services, provider of essential goods)?**

- Yes (1)
- No (2)
- Not applicable (4)

Display This Question:

If Are you an 'essential worker' (e.g., healthcare, law enforcement, emergency services, provider of... = Yes

Q4.7 **What type of essential work do you do?**

○ Healthcare (1)

○ Law enforcement (2)

○ Other emergency services (e.g., fire service) (3)

○ Provider of other essential goods or services (e.g., food supply, fuel, waste removal, internet, financial support, transport) (4)

Display This Question:

If Are you an 'essential worker' (e.g., healthcare, law enforcement, emergency services, provider of... = Yes

Q4.8 **Through your job, have you had known direct contact with COVID-19 patients?**

○ I have had direct contact with people who I knew at the time were **suspected of having** COVID-19 (2)

○ I have had direct contact with people who I knew at the time had **been diagnosed with** COVID-19 (1)

○ I found out later that people I had contact with were probable or confirmed COVID-19 cases but I did not know at the time (3)

○ I may have had contact with probable or confirmed COVID-19 cases (4)

○ To the best of my knowledge, I have not yet had contact with probable or confirmed COVID-19 cases (5)

Page Break

Display This Question:

If The following questions are about jobs and businesses during the COVID-19 lockdown. Do you ha... = I am self-employed

Or The following questions are about jobs and businesses during the COVID-19 lockdown. Do you ha... = I am a business owner

Q4.9 **Do you personally own or run a business whose reduction in turnover due to COVID-19 threatens the survival of your business?**

- Yes (1)
- No (2)

Display This Question:

If Do you personally own or run a business whose reduction in turnover due to COVID-19 threatens the... = Yes

Q4.10 **Have you personally had to make people in your organisation redundant or lose their jobs?**

- Yes (3)
- No (5)

Q4.11 **If there has been a reduction in your hours, have you applied (or has your employer applied on your behalf) for any of the following?**
Please select all that apply.

- [ ] COVID-19 wage subsidy (1)
- [ ] COVID-19 leave payment (2)
- [ ] Financial support for your business (6)
- [ ] Other government financial support (3)
- [ ] ⊗None of the above (4)
- [ ] ⊗Not applicable to me (5)
- [ ] ⊗I don't know (7)

Page Break

Display This Question:

If Do you personally own or run a business whose reduction in turnover due to COVID-19 threatens the... = Yes

Or Have your hours of paid work been drastically reduced as a result of the lockdown? = Yes

Or Have you lost your job (or jobs) as a result of the lockdown? = Yes

Q4.12 **How much has any reduction in your hours, losing your job, or loss of turnover in your business made it more difficult for you to meet basic living costs such as rent, mortgage payments, or food bills?**

- ○ A great deal (1)
- ○ A lot (2)
- ○ A moderate amount (3)
- ○ A little (4)
- ○ None at all (5)
- ○ Not applicable to me (6)

End of Block: 5. New Employment

Start of Block: 6. General Health

Q5.1 **Your general health**

The next few questions are about your general (physical) health.

Page Break

Q5.2 **How would you describe your general (physical) health?**

- ○ Poor (1)
- ○ Fair (2)
- ○ Good (3)
- ○ Very good (4)
- ○ Excellent (5)

Q5.3 **Over the past 5 years, have you had a medical condition that may make you more vulnerable to COVID-19 such as heart disease, COPD (difficulty breathing), weakened immunity, or cancer?**

- ○ Yes (1)
- ○ No (2)
- ○ Prefer not to say (3)

Q5.4 Do you have a family member who has a medical condition that may make them more vulnerable to COVID-19 such as heart disease, COPD (difficulty breathing), weakened immunity, or cancer?

- ○ Yes (1)
- ○ No (2)
- ○ Prefer not to say (3)

Q5.5 **Do you live with somebody, apart from a family member, who has a medical condition that may make them more vulnerable to COVID-19 such as heart disease, COPD (difficulty breathing), weakened immunity, or cancer?**

- Yes (1)
- No (2)
- Prefer not to say (3)

Q5.6 **Do you think you have had COVID-19?**

- Yes (1)
- No (2)
- Not sure (3)

Q5.7 **Have you been tested for COVID-19?**

- Yes (1)
- No (2)

Display This Question:

If Have you been tested for COVID-19? = Yes

Q5.8 **What were the results of this test?**

- Positive (1)
- Negative (2)
- Awaiting results (3)

Display This Question:

If What were the results of this test? = Positive

Q5.9 **Have you fully recovered from COVID-19?**

- Yes (1)
- No (2)

Q5.10 **Do you have any physical condition or disability that affects your ability to function (e.g., leave the house for essential goods or for physical activity) during the lockdown?**

- Yes (1)
- No (2)

End of Block: 6. General Health

Start of Block: 7. Alcohol use

Q6.1 **Alcohol and Smoking**

The following questions are about your alcohol intake and smoking since the start of the COVID-19 lockdown.

Q6.2

Q6.3

Q6.4 Using the above graphic as a guide, BEFORE **the lockdown, how many standard drinks would you have consumed in a typical 7 days?**

Please answer using a number

____

Q6.5 **How many standard drinks have you consumed in the last 7 days?**

Please answer using a number

____

End of Block: 7. Alcohol use

Start of Block: 8. Smoking

Q7.1 **How often do you CURRENTLY smoke tobacco cigarettes (either tailor-made or roll-your-own)?**

- ○ I smoke cigarettes every day (1)
- ○ I smoke cigarettes at least once a week, but not daily (2)
- ○ I smoke cigarettes less than once a week (3)
- ○ I am an ex-smoker (5)
- ○ I have never been a smoker (6)

Display This Question:

If How often do you CURRENTLY smoke tobacco cigarettes (either tailor-made or roll-your-own)? = I smoke cigarettes every day

Q7.2 **BEFORE the lockdown, about how many cigarettes did you smoke each day?**

Please answer using a number

____

Display This Question:

If How often do you CURRENTLY smoke tobacco cigarettes (either tailor-made or roll-your-own)? = I smoke cigarettes every day

Q7.3 **DURING the lockdown, about how many cigarettes do you smoke each day?**

Please answer using a number

____

Display This Question:

If How often do you CURRENTLY smoke tobacco cigarettes (either tailor-made or roll-your-own)? = I smoke cigarettes at least once a week, but not daily

Q7.4 **BEFORE the lockdown, about how many cigarettes did you smoke each week?**

Please answer using a number

____

Display This Question:

If How often do you CURRENTLY smoke tobacco cigarettes (either tailor-made or roll-your-own)? = I smoke cigarettes at least once a week, but not daily

Q7.5 **DURING the lockdown, about how many cigarettes do you smoke each week?**

Please answer using a number

____

End of Block: 8. Smoking

Start of Block: 9. Mental health

Q8.1 **How are you feeling?**

The next questions are about your mental health and wellbeing. We are interested in whether people's mental health or wellbeing may have been affected by recent COVID-19 events.

Some of these questions may seem a bit repetitive but they come from surveys that are

used all over the world. Please bear with us and answer them all.

Like all of the questions in the survey, your answers are completely confidential and anonymous, and will be used for research purposes only.

Q8.2 **DURING the lockdown**:

	**At no time** (1)	**Some of the time** (2)	**Less than half of the time** (3)	**More than half of the time** (4)	**Most of the time** (5)	**All of the time** (6)
I have felt cheerful and in good spirits (1)	o	o	o	o	o	o
I have felt calm and relaxed (2)	o	o	o	o	o	o
I have felt active and vigorous (3)	o	o	o	o	o	o
I woke up feeling fresh and rested (4)	o	o	o	o	o	o
My daily life has filled me with things that interest me (5)	o	o	o	o	o	o

Page Break

Q8.3 **The following question refers to your overall sleep quality for most nights both before and since the lockdown.**

Please think about the quality of your sleep overall, such as how many hours of sleep you got, how easily you fell asleep, how often you woke up during the night (except to go to the bathroom), how often you woke up earlier than you had to in the morning, and how refreshing your sleep was.

BEFORE the lockdown, how would you rate your sleep quality overall?

- ○ 1 (Terrible) (1)
- ○ 2 (2)
- ○ 3 (3)
- ○ 4 (4)
- ○ 5 (5)
- ○ 6 (6)
- ○ 7 (7)
- ○ 8 (8)
- ○ 9 (9)
- ○ 10 (Excellent) (10)

Q8.4 **<u>DURING the lockdown</u>, how would you rate your sleep quality overall?**

- 1 (Terrible) (1)
- 2 (2)
- 3 (3)
- 4 (4)
- 5 (5)
- 6 (6)
- 7 (7)
- 8 (8)
- 9 (9)
- 10 (Excellent) (10)

Page Break

Q8.5 **DURING the lockdown**, how often have you been bothered by the following?

	**Not at all** (1)	**Some days** (2)	**Most days** (3)	**Nearly every day** (4)
Feeling nervous, anxious, or on edge (1)	○	○	○	○
Not being able to stop or control worrying (2)	○	○	○	○
Worrying too much about different things (3)	○	○	○	○
Having trouble relaxing (4)	○	○	○	○
Being so restless that it's hard to sit still (5)	○	○	○	○
Becoming easily annoyed or irritable (6)	○	○	○	○
Feeling afraid as if something awful might happen (7)	○	○	○	○

Page Break

Q8.6 DURING the lockdown**, about how often have you felt tired out for no good reason?**

- ◯ None of the time (1)
- ◯ A little of the time (2)
- ◯ Some of the time (3)
- ◯ Most of the time (4)
- ◯ All of the time (5)
- ◯ Don't know (6)

Q8.7 DURING the lockdown**, about how often** have you felt **nervous?**

- ◯ None of the time (1)
- ◯ A little of the time (2)
- ◯ Some of the time (3)
- ◯ Most of the time (4)
- ◯ All of the time (5)
- ◯ Don't know (6)

Q8.8 <u>DURING the lockdown</u> **about how often** have you felt **so nervous that nothing could calm you down?**

- ◯ None of the time (1)
- ◯ A little of the time (2)
- ◯ Some of the time (3)
- ◯ Most of the time (4)
- ◯ All of the time (5)
- ◯ Don't know (6)

Q8.9 <u>DURING the lockdown</u>**, about how often** have you felt **hopeless?**

- ◯ None of the time (1)
- ◯ A little of the time (2)
- ◯ Some of the time (3)
- ◯ Most of the time (4)
- ◯ All of the time (5)
- ◯ Don't know (6)

Q8.10 <u>DURING the lockdown</u>**, about how often** have you felt **restless or fidgety?**

○ None of the time (1)

○ A little of the time (2)

○ Some of the time (3)

○ Most of the time (4)

○ All of the time (5)

○ Don't know (6)

Page Break

Q8.11 DURING the lockdown**, about how often** have you felt **so restless you could not sit still?**

- ○ None of the time (1)
- ○ A little of the time (2)
- ○ Some of the time (3)
- ○ Most of the time (4)
- ○ All of the time (5)
- ○ Don't know (6)

Q8.12 DURING the lockdown**, about how often** have you felt **depressed?**

- ○ None of the time (1)
- ○ A little of the time (2)
- ○ Some of the time (3)
- ○ Most of the time (4)
- ○ All of the time (5)
- ○ Don't know (6)

Q8.13 DURING the lockdown, **about how often** have you felt **that everything was an effort?**

- ◯ None of the time (1)
- ◯ A little of the time (2)
- ◯ Some of the time (3)
- ◯ Most of the time (4)
- ◯ All of the time (5)
- ◯ Don't know (6)

Q8.14 DURING the lockdown, **about how often** have you felt **so sad that nothing could cheer you up?**

- ◯ None of the time (1)
- ◯ A little of the time (2)
- ◯ Some of the time (3)
- ◯ Most of the time (4)
- ◯ All of the time (5)
- ◯ Don't know (6)

Q8.15 DURING the lockdown **about how often** have you felt **worthless?**

- ○ None of the time (1)
- ○ A little of the time (2)
- ○ Some of the time (3)
- ○ Most of the time (4)
- ○ All of the time (5)
- ○ Don't know (6)

Q8.16 DURING the lockdown w**hat have you found to be the main sources of stress or anxiety for you?**

Please tick all that apply

- ☐ Uncertainty regarding my health (1)
- ☐ Uncertainty regarding the health of my family or friends (2)
- ☐ Uncertainty regarding my finances (3)
- ☐ Uncertainty regarding my employment security (6)
- ☐ The wider consequences of COVID-19 (4)
- ☐ ⊗Not applicable (8)
- ☐ Something else (7) ____

Page Break

Q8.17 **Have you previously been diagnosed with a mental illness by a doctor or psychologist?**

- ○ Yes (1)
- ○ No (2)
- ○ Prefer not to say (3)

Display This Question:

If Have you previously been diagnosed with a mental illness by a doctor or psychologist? = Yes

Q8.18 **What diagnosis or diagnoses did they make?**

Please tick all that apply

- ☐ Depression (1)
- ☐ Bipolar disorder (2)
- ☐ Anxiety disorder (3)
- ☐ Personality disorder (4)
- ☐ Psychotic disorder (5)
- ☐ Alcohol or drug disorder (6)
- ☐ Other (7)
- ☐ ⊗Don't know (8)
- ☐ ⊗Prefer not to say (9)

Display This Question:

If Have you previously been diagnosed with a mental illness by a doctor or psychologist? = Yes

Q8.19 **DURING the lockdown, how is your mental health compared to usual?**

- ◯ Much worse than usual (1)
- ◯ Worse than usual (2)
- ◯ The same as usual (3)
- ◯ Better than usual (4)
- ◯ Much better than usual (5)
- ◯ Prefer not to say (6)

Page Break

Q8.20 **Important**

If any of these questions have caused you to feel distressed, or if you are struggling with your mental health, please free call or text 1737 or visit https://1737.org.nz to speak to a trained counseller. This also applies if you have any concerns for your friends, family or whānau.

Alternatively, you can call Depression.org.nz on 0800 111 757 or text 4202.

End of Block: 9. Mental health

Start of Block: 10. Previous trauma

Q226 **Have you ever been exposed to any of the following (aside from the current COVID-related events)?**

Please tick all that apply

- ▢ Childhood adversity (neglect, physical or sexual abuse) (1)
- ▢ Physical or sexual abuse after the age of 16 (2)
- ▢ Exposure to a traumatic event involving physical or sexual abuse to others (9)
- ▢ Natural disaster (e.g., fire, flood, earthquake) (3)
- ▢ Serious physical injury (e.g. car accident) (5)
- ▢ Serious illness (6)
- ▢ Other (please state) (7) ____
- ▢ ⊗ None of the above (8)

Page Break

End of Block: 10. Previous trauma

Start of Block: 11. Suicide

Q9.1 **Please note: The following questions ask about potentially sensitive topics.**

Like all of the questions in this survey, your answers are completely confidential and anonymous, and will be used for research purposes only.

But, if there are some questions you would prefer not to answer, just skip them.

Page Break

Q9.2 **BEFORE the lockdown, but during the previous 12 months, had you:**

	Yes (1)	No (2)	Prefer not to say (3)
**Seriously thought about ending your own life? (1)**	○	○	○
**Made plans to end your own life? (2)**	○	○	○
**Made an attempt to end your own life? (3)**	○	○	○

Q9.3 DURING the lockdown, have you:

	Yes (1)	No (2)	Prefer not to say (3)
**Seriously thought about ending your own life? (1)**	○	○	○
**Made plans to end your own life? (2)**	○	○	○
**Made an attempt to end your own life? (3)**	○	○	○

Q9.4 **If you feel distressed or feel like you want to talk about anything related to these issues, please call Lifeline on 0800 543 354 or text 4357.**

Alternatively, visit the Lifeline website at https://www.lifeline.org.nz/**.**

Page Break

End of Block: 11. Suicide

Start of Block: 12. Domestic violence

Q10.1 **Family violence**

The next two questions are about any incidents of family violence that have occurred in your household.

Remember, you are not obliged to answer these questions, but all responses are completely confidential and anonymous, and your responses will be used for research purposes only.

If family violence is curently an issue for your family/whānau **or friends, please contact one of the following organisations for assistance:**

Women's Refuge crisis line on 0800 733 843 - (24 hours)
Family violence information line on 0800 456 450
Emergency services on 111.

Page Break

Q10.2 **DURING the lockdown, have you experienced any of the following as a result of an action from a family/whānau member?**

Please tick all that apply, and in*clude threats made to you directly (face-to-face, phone, email, text), or via someone else*.

- ☐ Been frightened (8)
- ☐ Been insulted or abused (9)
- ☐ Been threatened with harm to you, your children or your pets (10)
- ☐ Been threatened with being hit, slapped or punched (5)
- ☐ Been threatened with a weapon or other object (6)
- ☐ Been slapped, punched or kicked (2)
- ☐ Been hit with a weapon or other object (3)
- ☐ Been touched sexually in a way you didn't like (4)
- ☐ Been forced to have sex when you didn't want to (1)
- ☐ ⊗None of these (7)
- ☐ ⊗Prefer not to say (11)

Q10.3 **<u>DURING</u> the lockdown, have you been a witness to any of the above in your 'bubble' ?**

- ○ Yes (1)
- ○ No (2)
- ○ Prefer not to say (3)

Q10.4 **Useful contacts for family violence-related incidents**

If family violence is currently an issue for your family/whānau or friends please contact one of the following organisations for assistance:

Women's Refuge
Call the crisis line on 0800 733 843 (24 hours)

'It's Not OK' family violence prevention
Call the information line on 0800 456 450

Rape Crisis
Call 0800 88 33 00 or visit http://www.rapecrisisnz.org.nz/

If you are presently in danger call the emergency services on 111.

End of Block: 12. Domestic violence

Start of Block: 13. Silver lining

Q11.1 **Positive aspects of COVID-19**

Have you experienced any 'silver linings' or positive aspects during the COVID-19 lockdown?

Please tick all that apply

- ☐ Yes, for me personally (11)
- ☐ Yes, for wider society (13)
- ☐ No (14)

Display This Question:

If Positive aspects of COVID-19 Have you experienced any 'silver linings' or positive aspects duri... = Yes, for me personally

Or Positive aspects of COVID-19 Have you experienced any 'silver linings' or positive aspects duri... = Yes, for wider society

Q11.2 **What are these silver linings, for you personally or for wider society?**

____

____

____

____

____

End of Block: 13. Silver lining

Start of Block: 14. Demographics

Q12.1 **Finally, a few questions about yourself**

Which of the following best describes your highest formal qualification?

- ○ No formal qualification (1)
- ○ High school qualifications (school certificate, NCEA, UE, Bursary) (2)
- ○ Certificate or diploma below Bachelor's level (3)
- ○ Bachelor's degree (4)
- ○ Post-graduate or higher qualification (5)

Q12.2 **What is your exact age (in years)?**

____

Q2.1
Which of these do you <u>most</u> identify with?

- ○ Male (1)
- ○ Female (2)
- ○ Gender diverse (3)

Display This Question:

If Which of these do you most identify with? = Gender diverse

Q12.3 **Are you?**

- ◯ Transgender female to male (1)
- ◯ Transgender male to female (2)
- ◯ Intersexed (3)
- ◯ Gender non-conforming (5)
- ◯ Genderqueer (6)
- ◯ Two-spirit (7)
- ◯ Third gender (8)
- ◯ Other (4) ____

Display This Question:

If Which of these do you most identify with? = Female

Q12.4

Are you pregnant?

- ◯ Yes (1)
- ◯ No (2)
- ◯ Don't know (3)

Q12.5 **What is the total income that you <u>yourself</u> received from all sources, before tax or any other deductions, over the last 12 months?**

○ Less than $10,000 (1)

○ $10,001 – $20,000 (2)

○ $20,001 – $30,000 (3)

○ $30,001 – $40,000 (4)

○ $40,001 – $50,000 (5)

○ $50,001 – $60,000 (6)

○ $60,001 – $70,000 (7)

○ $70,001 – $100,000 (8)

○ $100,001 – $150,000 (9)

○ $150,001 or more (10)

○ Prefer not to say (11)

Q12.6 **What is your postcode?**

____

End of Block: 14. Demographics

Start of Block: 15. Signposting

Q13.1 **Are there any comments you'd like to make about COVID-19, the lockdown or this survey? If so, please write them in the box below.**

____

____

____

Page Break

Q13.2
Remember

If you are feeling distressed by any of the content in this survey, think that these issues may be affecting family/whānau members or friends, or if you simply want more information, please note the following helplines and services.

Family violence

Women's Refuge
Call the crisis line on 0800 733 843 (24 hours)

'It's Not OK' family violence prevention
Call the information line on 0800 456 450

Rape Crisis
Call 0800 88 33 00 or visit http://www.rapecrisisnz.org.nz/

If you are in danger, call the emergency services on 111.

Depression or suicide

Lifeline
Call 0800 543 354 or text 4357 or on the web at https://www.lifeline.org.nz/.

NZ free and confidential counselling
Call or text 1737 or visit https://1737.org.nz/

Depression.org.nz
Call 0800 111 757, text 4202, or visit depression.org.nz.

COVID-19 information
Call the Ministry of Health Healthline on 0800 611 116 for advice,
or visit https://covid19.govt.nz/ for up-to-date and accurate information on COVID-19.

Page Break

Q13.4 **Thank you for taking part in our survey.**

Please click 'Next' to submit your answers.

End of Block: 15. Signposting
