# Supplementary material for: Thriving in a pandemic: Determinants of excellent wellbeing among New Zealanders during the 2020 COVID-19 lockdown; a cross-sectional survey
Source: PLoS One. 2022 Mar 3;17(3):e0262745. doi: 10.1371/journal.pone.0262745 (PMC8893611; doi:10.1371/journal.pone.0262745)
Supplement: Attachment — Submitted filename: Response to reviewers PLOS-1.docx [file pone.0262745.s003.docx]

**13.12.21**

**Response to reviewers**

Dear Dr Joshi

Thank-you for the opportunity to revise *Thriving in a pandemic: determinants of excellent wellbeing among New Zealanders during the 2020 COVID-19 lockdown; a cross-sectional survey.*

**Response to editorial questions**

1. We have reviewed the style requirements and believe these are met.
2. We have included the Inclusivity of global research questionnaire as a Supporting Information document.
3. We have amended our acknowledgements statement to make the relationship with Dynata more clear. We were grateful for their support of the project but they did not provide any funding. The study was completed without funding so we do not need to update the Funding Statement.
4. Data availability statement; we will place the data in a repository if accepted for publication but have not done so at this stage.
5. Captions for Supporting Information files are now included.
6. Supporting Information S1 file has now been uploaded.

We thank Reviewers 1 and 2 for their constructive comments. We believe their feedback has improved the paper. Our responses to their comments follows.

**Reviewer 1**

1. We have expanded on our original explanation relating to the merging of the datasets in the methods. We have also provided additional comment in the limitations section of the discussion to provide context to the decision to merge data.
2. We have expanded the introduction as suggested to provide better context to our research. We have also referenced the Rohr paper in the discussion. The tracked changes version highlights the areas of change.

**Reviewer 2**

1. Title: We have added ‘cross-sectional survey’ to the study title as per the CROSS checklist.
2. Abstract: Methods; we have added details of the design, sample size, and survey administration to the methods. We have also provided the lockdown dates.
3. Abstract: Results; we have added the number of respondents (303). We have also specified increasing age (this finding was part of the multivariable analysis) and detailed the level of education (certificate/diploma level or under). We have also clarified that the findings relate to multivariable analysis and provided p values.
4. Abstract: Conclusion; we have removed ‘substantial minority’ and replaced this with the more accurate ‘Nine percent’.
5. We have merged the citations as suggested.
6. Methods: Sample; the sample size was 3487 as reported but we made an error in reporting the Dynata sample (this should be 2010). We have corrected this in the results. Nineteen participants did not provide full WHO-5 data and were excluded from the analysis. A total of 106 participants had missing data for some of the other variables. These participants are included in the descriptive and univariable analyses but are excluded from the multivariable analysis. We have clarified this in the methods section and results section.
7. Results: Total participants; see above for details.
8. Results: Missing values now included in Table 1.
9. Results: We have amended the reporting of % figures to include the number of participants.
10. We have now included the SD for the mean WHO-5 score
11. Results: Line 238 signposts Table 1 in the text
12. Results: We have added the p-values for the reported statistics.
13. Results: We have clarified that the lower education level is certificate/diploma level qualification or less.
14. Results: We have now provided the relevant statistics when associations are reported.
15. Results: Table 2. We have provided more explanation in the methods. The data we report from the NZGS is not in the public domain. Further analysis of the NZGSS data was required to report excellent wellbeing. We therefore believe that this data belongs in the results section.
